# Predictive factors and risk analysis of recurrent laryngeal nerve invasion in papillary thyroid carcinoma ≤ 1 cm

**DOI:** 10.20945/2359-3997000000537

**Published:** 2023-01-17

**Authors:** Fan Yang, Jianhong Wang, Yuansheng Rao, Yanjun Feng, Lingzhao Meng, Jugao Fang

**Affiliations:** 1 Capital Medical University Beijing Anzhen Hospital Department of Otorhinolaryngology Head and Neck Surgery Beijing China Beijing Anzhen Hospital, Capital Medical University, Department of Otorhinolaryngology Head and Neck Surgery, Beijing, China; 2 Capital Medical University Beijing Tiantan Hospital Department of Otorhinolaryngology Head and Neck Surgery Beijing China Beijing Tiantan Hospital, Capital Medical University, Department of Otorhinolaryngology Head and Neck Surgery, Beijing, China; 3 Capital Medical University Beijing Tongren Hospital Department of Otorhinolaryngology Head and Neck Surgery Beijing China Beijing Tongren Hospital, Capital Medical University, Department of Otorhinolaryngology Head and Neck Surgery, Beijing, China

**Keywords:** Papillary thyroid carcinoma, recurrent laryngeal nerve, surgical management, recurrence, predictive factor

## Abstract

**Objective:**

The recurrent laryngeal nerve (RLN) may be involved by papillary thyroid carcinoma ≤ 1 centimeter (PTC ≤ 1 cm). Current study investigated the predictive factors of RLN invasion in PTC ≤ 1 cm, the risk factors of disease recurrence in RLN invaded cases and the results of surgical management for RLN invasion.

**Materials and methods:**

Data of 374 PTC ≤ 1 cm patients were retrospectively collected. We performed univariate and multivariate analysis to identify predictive factors of RLN invasion and risk factors of disease recurrence. The abilities of factors in predicting RLN invasion were evaluated. Surgical outcomes and recurrence free survival (RFS) of patients were analyzed.

**Results:**

A total of 28 patients suffered RLN invasion, among which seven had disease recurrence. Preoperative vocal cord palsy (VCP), gross extrathyroidal extension, larger tumor size and tumor on the dorsal side of thyroid were verified as predictive factors of RLN invasion. RLN involved patients had poorer RFS, but better than those who also had upper-aerodigestive tract invasion. Upper-aerodigestive tract invasion, lateral neck lymph nodes metastasis (LNM) and BRAF V600E mutation were independent risk factors of disease recurrence in RLN invaded cases. Tumor shaving showed better RLN function preservation without increasing recurrent risk.

**Conclusions:**

Current study confirmed the rarity of RLN invasion in PTC ≤ 1 cm. Various aggressive features were verified as predictive factors of RLN invasion. Tumor shaving showed superiority in preserving nerve function without increasing recurrent risk. Special attentions should be paid for disease recurrence when RLN invasion accompanied by upper-aerodigestive tract invasion, lateral neck LNM or BRAF V600E mutation.

## INTRODUCTION

The incidence of differentiated thyroid carcinoma (DTC) increases rapidly in recent decades with the development of modern medical technology and the adoption of imaging modalities (1,2), among which most were papillary thyroid carcinoma ≤ 1 centimeter (PTC ≤ 1 cm) with the characteristics as indolent behavior and favorable prognosis (3,4). On the contrary, despite the tiny tumor size, some cases still have poorer quality of life when adjacent structures were invaded, resulting in corresponding symptoms and impaired survival outcomes (5,6). The recurrent laryngeal nerve (RLN) is one of the most common structures to be invaded by a primary thyroid tumor or metastatic lymph node (7).

Although preoperative examinations, for instance laryngoscopy, are routinely used to identify preoperative function of the RLN, it is insufficient to detect RLN invasion, because not all patients with RLN invasion will develop vocal cord palsy (VCP) (8,9). Therefore, it is necessary to determine potential factors that may suggest RLN invasion, or affect survival outcomes with regard to RLN invasion; however, to our knowledge, few articles have investigated these on PTC ≤ 1 cm. The objective of the study was to identify and evaluate predictive factors of RLN invasion in patients with PTC ≤ 1 cm, as well as independent risk factors of disease recurrence. The study findings would be helpful for thyroid surgeons, aiding determination of the proper indication in terms of surgical management and postoperative surveillance.

## MATERIALS AND METHODS

### Study patients

The demographic and clinicopathological data of patients with PTC ≤ 1 cm who underwent surgery at our institution between 2012 and 2018 were retrospectively collected. The inclusion criteria were PTC with diameters no more than 1cm as confirmed by pathological examination. The exclusion criteria were as follows: age under 18 years, any history of thyroid or neck surgery, any history of radiation exposure, family history of thyroid cancer, and presence of other tumors or missing clinical data that may influence the statistical results. Informed consent was obtained from all participants included in the study. The study was conducted in accordance with the Declaration of Helsinki (as revised in 2013). The study was approved by the ethical review board of Beijing Anzhen Hospital (No. 2021099X) and individual consent for this retrospective analysis was waived.

### Disease management and follow-up

All study patients underwent preoperative ultrasonography evaluation of thyroid nodules and potential lymph nodes metastasis (LNM) in the central or lateral compartment of neck. RLN function of each patient was evaluated by laryngoscopy before and after surgery. Any reduced cord movement was recorded as VCP. Computed tomography (CT) was also performed in patients with potential invasion to the larynx, trachea, esophagus, or RLN. After providing a full explanation of the potential risks and benefits of surgical treatment, patients’ decision on whether to undergo surgery was considered. Unilateral thyroidectomy plus isthmectomy combined with prophylactic central compartment neck dissection was performed by the same experienced surgical team. Total thyroidectomy was performed for cases with unilateral or bilateral multifocal tumors, multiple bilateral thyroid nodules, LNM and extrathyroidal extension. Patients with suspicious preoperative lateral neck LNM or intraoperative pathologically confirmed lateral neck LNM also underwent lateral neck dissection involving at least levels II-IV. Radioactive iodine and thyroid-stimulating hormone-suppressive hormonal therapy were recommended to postoperative patients according to the established guidelines (10). As for surgical managements of the RLN that involved by primary tumor or metastatic lymph node, if possible, the lesion tissue was meticulously shaved off the nerve under magnification by surgical loupes in order to maximally remove the lesion while preserving the function of RLN. If the RLN was invaded extensively, the RLN was resected and an anastomosis between the two segments of RLN was made, if feasible. The laryngoscopy examination was performed after surgery and during every return visit, if postoperative VCP or any complaints of voice change were identified. Patients with postoperative VCP more than 1 year were considered to have permanent paralysis. Patients who developed hypocalcemia were started on oral calcium and vitamin D replacement and intravenous calcium gluconate for significant symptoms. Permanent hypoparathyroidism was considered in patients with low total calcium concentrations that required calcium supplementation for more than six months. Assessment for recurrence was performed including manual palpation, ultrasonography, and serum thyroglobulin level. CT, FDG-PET-CT, or whole-body scans were conducted when appropriate. Recurrence was confirmed by ultrasound-guided biopsy or histopathological examination after secondary surgery.

### Statistical analyses

Continuous variables were represented as the mean ± standard deviation (*n* ± *sd*) and analyzed using the Student's t-test or Mann-Whitney U test. Categorical variables were tested with Chi-square test or Kruskal-Wallis test. Variables found to be significantly different between groups in the univariate analysis were included in the logistic regression analysis. The ability of factors in predicting RLN invasion in PTMC was evaluated, the values of sensitivity, specificity, positive predictive value, negative predictive value and positive/negative likelihood ratio were calculated. Survival data regarding the recurrence were analyzed using the Kaplan-Meier method and compared with the log-rank test. Cox proportional hazards model was used to estimate the hazard ratio of factors related to recurrence. A two-tailed *p* < 0.05 was determined as statistically significant. SPSS 25.0 (SPSS, Inc., Chicago, IL, USA) and MedCalc^®^ Software 19.8 (MedCalc Software Ltd, Ostend, Belgium) was used for all statistical analyses. The recurrence free survival (RFS) curves were plotted using R (v3.5.1) with packages *survival, survminer* and *ggplot2*.

## RESULTS

### Baseline characteristics

A total of 374 patients with 565 tumors were included in this study, out of whom 369 (98.7%) patients with 559 (98.9%) tumors were classic PTC, three (0.8%) patients with four (0.7%) tumors were follicular variant PTC, and two (0.5%) patients with two (0.4%) tumors were encapsulated variant PTC. Detailed demographic and clinicopathological information are shown in Table 1. As well, unilateral thyroidectomy plus isthmectomy were performed on 222 (59.4%) patients, and total thyroidectomy were performed on 152 (40.6%) patients. All of the patients underwent central neck dissection, of whom 43 (11.5%) also received lateral neck dissection.

**Table 1:** Basic information and results of univariate analysis between patients with/without RLN invasion

Characteristics		All n (%)	RLN invasion		
			RLN-n (%)	RLN+n (%)	*p* value
Sex					0.249^b^
	Female	275 (73.5%)	257 (74.3%)	18 (64.3%)	
	Male	99 (26.5%)	89 (25.7%)	10 (35.7%)	
Age					0.572^b^
	<55 years	277 (74.1%)	255 (73.7%)	22 (78.6%)	
	≥55 years	97 25.9%)	91 (26.3%)	6 (21.4%)	
Body mass index					0.260^b^
	<25	216 (57.8%)	197 (56.9%)	19 (67.9%)	
	≥25	158 (42.2%)	149 (43.1%)	9 (32.1%)	
Hashimoto thyroiditis					0.488^b^
	Yes	57 (15.2%)	54 (15.6%)	3 (10.7%)	
	No	317 (84.8%)	292 (84.4%)	25 (89.3%)	
Multifocality					**0.035** b
	Yes	144 (38.5%)	128 (37.0%)	16 (57.1%)	
	No	230 (61.5%)	218 (63.0%)	12 (42.9%)	
Bilateral tumor					0.466^b^
	Yes	86 (23.0%)	78 (22.5%)	8 (28.6%)	
	No	288 (77.0%)	268 (77.5%)	20 (71.4%)	
Central neck LNM					**<0.001** b
	Yes	145 (38.8%)	124 (35.8%)	21 (75.0%)	
	No	229 (61.2%)	222 (64.2%)	7 (25.0%)	
Lateral neck LNM					**<0.001** b
	Yes	36 (9.6%)	26 (7.5%)	10 (35.7%)	
	No	338 (90.4%)	320 (92.5%)	18 (64.3%)	
Preoperative VCP					**<0.001** b
	Yes	11 (2.9%)	2 (0.6%)	9 (32.1%)	
	No	363 (97.1%)	344 (99.4%)	19 (67.9%)	
Extrathyroidal extension					**0.01** c
	None	295 (78.9%)	279 (80.6%)	16 (57.1%)	
	Micro ETE	18 (4.8%)	16 (4.6%)	2 (7.1%)	
	Gross ETE	61 (16.3%)	51 (14.7%)	10 (35.7%)	
BRAF V600E mutation					0.069^b^
	Yes	249 (66.6%)	226 (65.3%)	23 (82.1%)	
	No	125 (33.4%)	120 (34.7%)	5 (17.9%)	
Average tumor size (cm)		0.60±0.28	0.59±0.27	0.82±0.31	**<0.001** d
Average tumor number		1.51±0.75	1.49±0.73	1.79±0.92	0.055^c^
Number of central neck lymph nodes excised		7.04±5.31	6.88±4.90	9.00±8.82	0.561^c^
Number of central neck LNM		1.13±2.00	1.00±1.83	2.86±3.04	**<0.001** c
Number of lateral neck lymph nodes excised		32.40±21.15	32.74±22.05	31.50±19.48	0.865^d^
Number of lateral neck LNM		5.51±4.23	4.32±3.19	8.58±5.13	**0.002** d
Tumor depth^a^					**<0.001** b
	Ventral side of thyroid	310 (54.9%)	304 (56.6%)	6 (21.4%)	
	Dorsal side of thyroid	255 (45.1%)	233 (43.4%)	22 (78.6%)	
Tumor location^a^					0.125^c^
	Upper 1/3	205 (36.3%)	189 (35.2%)	16 (57.1%)	
	Middle 1/3	173 (30.6%)	167 (31.1)	6 (21.4%)	
	Lower 1/3	179 (31.7%)	173 (32.2%)	6 (21.4)	
	Isthmus	8 (1.4%)	8 (1.5%)	0 (0%)	

a Based on the characteristics of tumors.

b Chi-square test.

c Kruskal-Wallis test.

d Student's t test.

RLN (+/-): with/without recurrent laryngeal nerve invasion; LNM: lymph node metastasis; VCP: vocal cord palsy.

In this study, 28 (7.5%) patients were found to have RLN invasion, 19 (19/28, 67.9%) cases were tumor direct invasion and nine (9/28, 32.1%) were LNM; of these patients, 20 (20/28, 71.4%) cases underwent tumor shaving and eight (8/28, 28.6%) cases underwent RLN resection respectively. Univariate analysis was conducted between the group with (n = 28) and without (n = 346) RLN invasion: when compared between the distribution of patients with/without RLN invasion, significant differences were observed in variables including multifocality, central neck LNM, lateral neck LNM, preoperative VCP, extrathyroidal extension, and number of central and lateral neck LNM; when compared between the characteristics of tumors (based on the 565 tumors) in cases with/without RLN invasion, significant differences were observed in variables including average tumor size and tumor depth (Table 1).

There were no significant differences in sex, age, body mass index, Hashimoto thyroiditis, bilateral tumor, with/without BRAF V600E mutation, tumor number, total number of lymph nodes excised and tumor location in thyroid when compared between patients with/without RLN invasion.

### Predictive factors of RLN invasion in PTC ≤ 1 cm

As mentioned, RLN invasion was more often observed in patients with multifocal tumors, central and lateral neck LNM, preoperative VCP, gross extrathyroidal extension, larger tumor size, high volume of LNM and dorsal side tumor. Further multivariate analysis by logistic regression with following variables was conducted in order to screening factors in predicting RLN invasion: multifocality, preoperative VCP, gross extrathyroidal extension, tumor size, and tumor depth. The results showed that RLN invasion in PTC ≤ 1 cm was strongly associated with preoperative VCP, gross extrathyroidal extension, larger tumor size and tumor on the dorsal side of thyroid (Table 2).

**Table 2:** Multivariate analysis and predictive ability analysis in factors associated with RLN invasion

Characteristics	OR	95% CI	*p* value^a^	Sensitivity	Specificity	PPV	NPV	LR+	LR-
Preoperative VCP	27.484	4.197-179.990	0.001	17.9	99.4	70.75	93.7	30.89	0.83
Gross extrathyroidal extension	2.96	1.244-7.046	0.014	42.9	80.6	15.2	94.6	2.21	0.71
Large tumor size	12.533	3.545-44.303	<0.001	82.1	52.9	12.4	97.3	1.74	0.34
Tumor on the dorsal side of thyroid	4.784	1.909-11.989	0.001	78.6	56.6	8.7	98	1.81	0.38
Multifocality	1.869	0.818-4.270	0.138	-	-	-	-	-	-

a Logistic regression analysis.

OR: odds ratio; CI: confidence interval; VCP: vocal cord palsy; PPV: positive predictive value; NPV: negative predictive value; LR+/-: positive/negative likelihood ratio.

We evaluated the predictive abilities of the characteristics that strongly associated with RLN invasion. As shown in Table 2, larger tumor size showed the highest sensitivity and preoperative VCP showed the highest specificity. All of the four characteristics revealed favorable negative predictive value while preoperative VCP had the best positive predictive value. In addition, patients with preoperative VCP were 30.89 times more likely to have RLN invasion, followed by gross extrathyroidal extension LNM and tumor on the dorsal side of thyroid.

### Surgical complications and postoperative recovery

**Figure 1:**
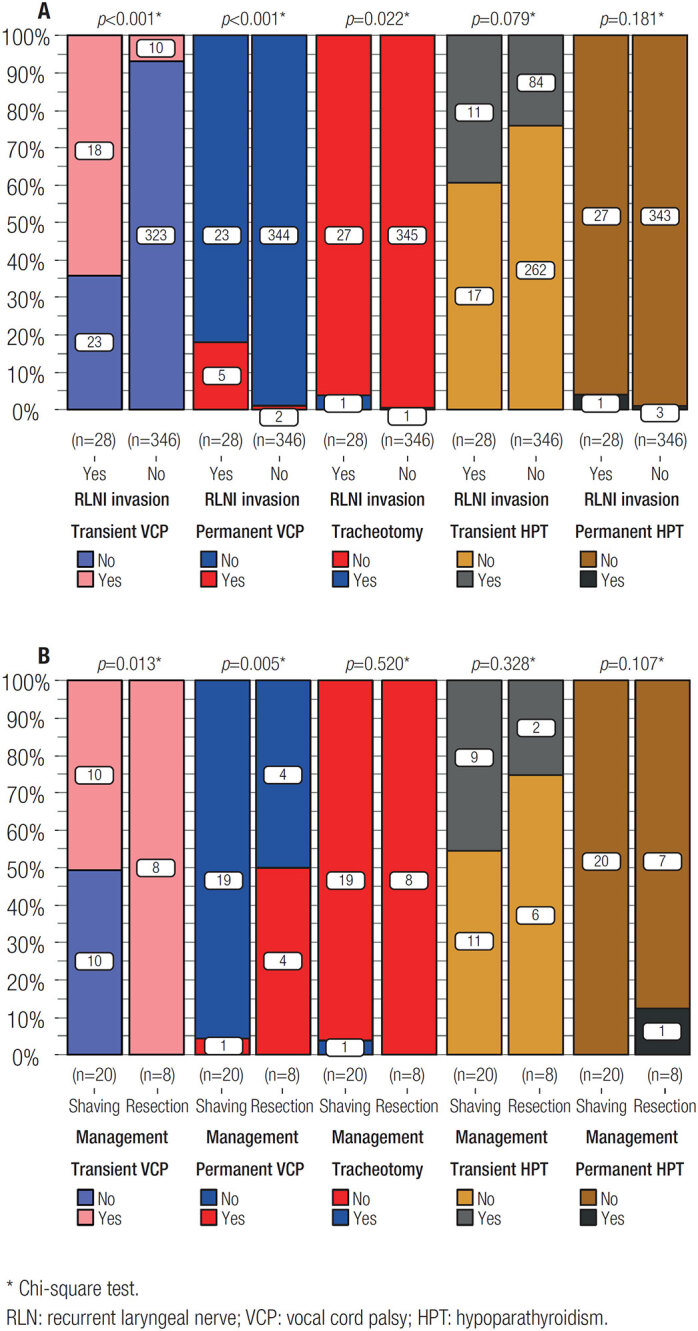
Comparison results of surgical complications. (**A**): Compared between patients with or without RLN invasion. (**B**): Compared between tumor shaving and nerve resection.

Of all patients received surgery, transient postoperative VCP was observed in 28 patients in all, and 21 of them finally restored vocal cord function. Those with RLN invasion suffered much more transient and permanent postoperative VCP. Prophylactic tracheotomy was implemented to two patients (the proportion in the RLN invasion group was significantly higher than that without RLN invasion) and they all got decannulated after compensatory of vocal cord function finally. Transient hypoparathyroidism occurred in 95 patients; four patients were eventually determined as permanent hypoparathyroidism (Figure 1).

The comparison between the two management methods toward RLN invasion showed RLN resection technique resulted in much higher proportion of transient and permanent postoperative VCP than RLN shaving technique; while there were non-significant differences in the incidence of tracheotomy and hypoparathyroidism between the two management methods (Figure 1).

### Risk factors of disease recurrence in PTC ≤ 1 cm with RLN invasion

**Figure 2:**
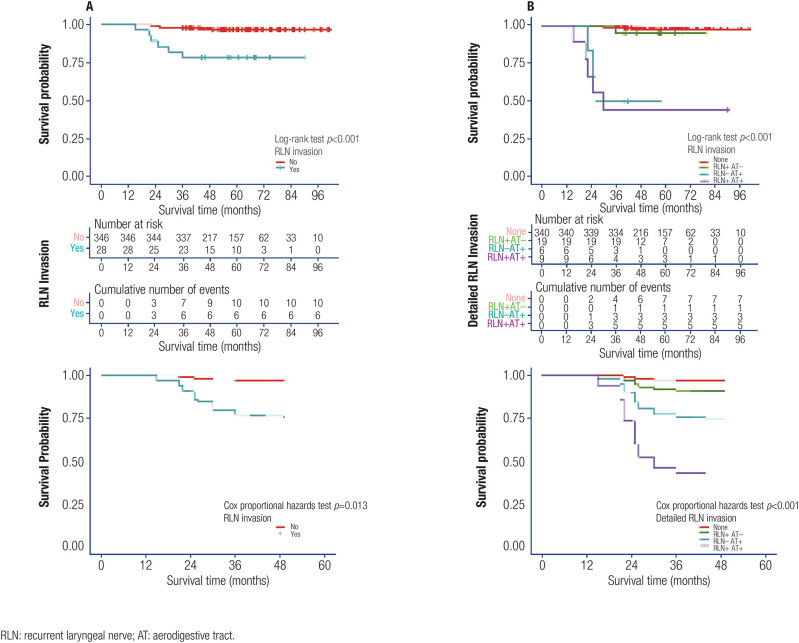
Results of disease recurrence free survival analysis among all patients. From up to bottom showed the RFS survival curves, number at risk, cumulative number of events and RFS survival curves adjusted for age, sex and body mass index. (**A**): Compared between patients with/without RLN invasion. (**B**): Compared among patients without RLN and upper-aerodigestive tract invasion, with only RLN invasion, with only upper-aerodigestive tract invasion and with both RLN and upper-aerodigestive tract invasion.

During the follow-up period (averaged 57.7 ± 17.5 months), primary or cervical lymphatic recurrence was observed on 12 (3.2%) patients, including seven with RLN invasion. As shown in Figure 2A, patients with RLN invasion had much unfavorable RFS outcomes than those without RLN invasion. Similar result was observed after adjusted for age, sex and BMI. Further analysis revealed that patients with both RLN and upper-aerodigestive tract invasion had the worst RFS outcome, followed by those with only upper-aerodigestive tract invasion; the RFS outcome of patients with only RLN invasion was worse than those without any invasion to RLN or upper-aerodigestive tract, but much better than those with upper-aerodigestive tract invasion, no matter if RLN was invaded (Figure 2B). We also compared the RFS between the two types of surgical management (shaving *vs*. resection) for RLN invasion and observed no statistical difference between them (Log-rank test *p* value of 0.473).

Of the 28 RLN invaded cases, seven patients also had esophageal invasion, three had laryngotracheal invasion and two had gross strap muscle invasion. As for LNM, 21 patients had central neck LNM, among which ten patients were also observed with lateral neck LNM. To further identify the potential risk factors for recurrence in patients with RLN invasion, we performed univariate and multivariate analysis with variables including sex, age, body mass index, Hashimoto thyroiditis, multifocality, gross strap muscle invasion, central and lateral neck LNM, esophageal invasion, laryngotracheal invasion, BRAF V600E mutation, RLN invasion type (direct invasion or LNM), tumor depth and location. Consequently, the independent risk factors that adversely affected RFS in PTC ≤ 1 cm with RLN invasion were: esophageal invasion, laryngotracheal invasion, lateral neck LNM and with BRAF V600E mutation (Table 3, Supplementary Table 1).

**Table 3:** Multivariate analysis of independent risk factors of RFS for patients with RLN invasion

Characteristics	HR	95% CI	p value^a^
Esophageal invasion	8.015	1.442-44.543	0.017
Laryngotracheal invasion	7.688	1.362-43.189	0.021
Lateral Neck LNM	11.57	1.299-95.805	0.028
BRAF V600E mutation	8.288	1.010-67.977	0.049

a Cox proportional hazards model.

HR: hazard ratio; CI: confidence interval.

**Supplementary Table 1:** Univariate analysis of RFS risk factors for patients with RLN invasion.

Characteristics		HR	95% CI	p value^a^
Sex				
	Female	Reference	-	-
	Male	.278	0.033-2.311	0.236
Age				
	< 55 years	Reference	-	-
	≥ 55years	.032	0.0-47.587	0.355
Body mass index				
	< 25	Reference	-	-
	≥ 25	1.756	0.392-7.869	0.462
Hashimoto thyroiditis				
	No	Reference	-	-
	Yes	0.041	0.0-755.584	0.523
Multifocality				
	No	Reference	-	-
	Yes	1.014	0.227-4.537	0.985
Gross strap muscle invasion				
	No	Reference	-	-
	Yes	2.064	0.247	17.245
Central neck LNM				
	No	Reference	-	-
	Yes	0.921	0.178-4.758	0.922
Lateral neck LNM				
	No	Reference	-	-
	Yes	6.036	1.160-31.402	**0.033**
Esophageal invasion				
	No	Reference	-	-
	Yes	10.505	2.008-54.966	**0.005**
Laryngotracheal invasion				
	No	Reference	-	-
	Yes	6.234	1.180-32.936	**0.031**
BRAF V600E mutation				
	No	Reference	-	-
	Yes	6.179	1.179-32.386	**0.031**
RLN invasion type				
	Direct invasion	Reference	-	-
	LNM	1.349	0.261-6.958	0.721
Tumor depth				
	Ventral side of thyroid	Reference	-	-
	Dorsal side of thyroid	0.932	0.180-4.818	0.933
Tumor location				
	Upper 1/3	Reference	-	-
	Middle 1/3	1.208	0.221-6.601	0.828
	Lower 1/3	0.668	0.075-5.977	0.718

a: Cox proportional hazards model, only those *p* < 0.05 were further included for multivariate analysis; HR, hazard ratio; CI, confidence interval, LNM, lymph node metastasis; RLN, recurrent laryngeal nerve; VCP, vocal cord palsy.

## DISCUSSION

The existing management strategy for locally invasive thyroid cancer aims to achieve a completely resection of tumor while maintaining structural function and patients’ quality of life (11,12). As reported, local invasion occurs in approximately 13%-15% of cases in DTC, and the RLN is one of the most commonly involved structures (13–15). Anatomically, the left RLN ascends posteromedial to the thyroid gland in the tracheoesophageal groove, after entering the neck; where the right RLN runs in a more lateral and anterior location in the neck, then coursing posteriorly and medially before entering into the larynx (16). There are few researches focused on the impact of RLN invasion in PTC ≤ 1 cm, so we conducted a retrospectively study and identified predictive factors for RLN invasion in PTC ≤ 1 cm, as well as risk factors for disease recurrence in RLN invaded patients.

The first thing that needs to be emphasized is that PTC ≤ 1 cm, or once known as papillary thyroid microcarcinoma (PTMC), is no longer being considered as an independent type of thyroid neoplasm in the latest WHO report, since the identification of genetic alterations in thyroid carcinogenesis has shifted the classification of thyroid cancer (17). We included BRAF V600E mutation, which is the most representative molecular signature in PTC, as a variable in this study and revealed similar results as once widely reported: BRAF V600E mutation occupied up to 2/3 of all cases and is of great potentials in predicting tumor recurrence (18). On the other hand, BRAF V600E mutation did not predict RLN invasion in PTC ≤ 1 cm, and we reckon that the very characteristic of the tumor itself may have much significant impacts on the probability of RLN invasion.

Up to two third of locally invasive DTC patients suffered RLN invasion directly from thyroid primary tumor or from paratracheal lymphadenopathy (13,19,20). In current study, the incidence of RLN invasion was 7.5%, much lower than those in DTC, but higher than a recent study by Chen and cols. who observed a RLN invasion rate of 3.2% (21). We speculate this could be attributed to different sample selection. RLN invasion is easy to be diagnosed via endoscopic examination when hoarseness or VCP occurred; but a conclusive diagnosis in cases without paralysis is reportedly challenging since imaging procedures of the central neck compartment suffer from low sensitivity (8). We recorded near one-third of RLN-invaded patients showed preoperative VCP, which added to the accumulating evidence that preoperative VCP is a strong predictor of RLN involvement, not only in DTC but in PTC with small diameters (15,22,23). Notably, not all preoperative VCP were caused by tumor infiltration, and not all RLN invasion will inevitably lead to VCP. Therefore, preoperative clinical evaluation including preoperative laryngoscopy and assessment of RLN risk is essential to formulating a surgical plan and providing appropriate patient counseling.

The prevalence of LNM and extrathyroidal extension has been known to associate with the tumor size (24,25). In our analysis, tumor size was confirmed as a predictor for RLN invasion. This partly supported those higher chances of peripheral structure involvement and corresponding symptoms, including VCP, were more likely to be observed in larger thyroid tumors (26,27). Similar to the results of Zheng and cols. (28), more evidence is needed to support the correlation between the vertical location of the tumor and RLN invasion in current study; while RLN invasion was much commonly presented in cases where tumor located on the dorsal side of thyroid gland as compared to those on the ventral side, which indicated that features of posterior tumor should be given more attentions to the possibility of RLN invasion, even in the absence of VCP (29).

Upper-aerodigestive tract has anatomic proximity to the thyroid gland, thus is susceptible to extrathyroidal thyroid tumors. In most DTC patients with RLN invasion, the upper-aerodigestive tract were often invaded, which increased the difficulty of complete surgical resection and always led to impaired prognosis (20,30). Current study in PTC ≤ 1 cm corroborated with previous reports that gross extrathyroidal extension is strongly associated with RLN invasion (11, 21). Similarly, we identified the adverse impact of upper-aerodigestive tract invasion on RFS: patients with upper aerodigestive tracts invasion suffered more disease recurrences no matter if RLN was invaded. These results were in line with those reported by Kim and cols. and Shindo and cols. (6,30). On the other hand, Ito and cols. (31) demonstrated that RLN invasion alone may not affect long-term survival in PTC if upper aerodigestive tracts were not involved; while Lang and cols. (32) observed worse cancer specific survival outcomes in RLN invaded PTC cases. Here we found that patients with single RLN invasion still showed worse RFS than those without any extrathyroidal extension. These differences can be attributed to different sample selection or follow-up times, however the influences induced by RLN invasion may still be controversial. We hope current study could inspire further researches since loss of RLN function causes multiple symptoms and may result in considerable deterioration of the patients’ quality of life, even if it may not affect patients’ prognosis.

In addition to extrathyroidal extension, some cases in this study exhibited other aggressive behaviors, for example LNM (33). The LNM in thyroid cancer often occurs in the central neck, so that RLN could also be infiltrated (28). More importantly, a fair number of patients suffered asymptomatic RLN invasion by LNM until detected in thyroid surgery (8). Intraoperative detected suspicious lymph nodes around the RLN should be adequately visualized and dissected to check for metastasis since only one of the nine patients with RLN invaded by LNM showed preoperative VCP in current study. Occult lymphatic metastasis in cN0 PTC patients has been reported constantly and prophylactic central neck dissection was recommended for the purpose of accurate staging and follow-up management, and these became strong rationale for performing prophylactic central neck dissection on all patients at the time when patients receiving surgeries in this study (10,34). However the recent years have witnessed a rapid growth in the controversial of prophylactic central neck dissection on low-risk patients for it did not show superior in extend patients’ life but increased the incidence of surgical complications (35–37). Therefore we believe prophylactic central neck dissection should follow the principle of individualization and should be applied with more caution. Depending on the survey, the recurrence of lymphatic metastasis accounted for nearly 80% of the total recurrences in PTMC and lateral neck LNM has been verified as an independent risk factor for locoregional recurrence (38–40). Similarly, we confirmed that among those with RLN invasion, the occurrence of lateral neck LNM led to increased risk of tumor recurrence. In all, extrathyroidal extension and LNM commonly denoted a more aggressive tumor behavior, thus attentions should be paid to the RLN and surrounding structures during surgery for thyroid cancer, especially for those who are suspected to have peripheral structure involvement during preoperative examination (41).

Visual identification of the RLN during surgery is critical, and as such, surgeons must have intimate familiarity with its surgical anatomy. Voice impairment after thyroid surgery may engender severe emotional or psychological distress as the patient comes to terms with the surgical outcome. We recorded an overall transient postoperative VCP rate of 11%, the prevalence of both transient and permanent VCP were much common among patients with RLN invasion in this study, which resembled several previous studies (42–44). Moreover, there was no difference of RFS between the two methods dealing with tumor involvement on RLN, but shaving technique showed much better restoration of postoperative VCP, and these was also in accordance with some researches that conducted on PTC (20,21,32). Kihara and cols. (45) found that nearly 80% of the RLN nerve cross-section sample was mainly composed of perineural connective tissue that surrounding a small component of nerve fibers, suggested that in patients with preoperative VCP, the RLN may not be completely invaded but can still stimulate intrinsic laryngeal muscles to prevent atrophy of the vocal cords. They attempted to resect perineural tissue only, preserving the core portion of the nerve as far as possible. Consequently, about 83% patients restored nearly normal phonatory function. Other studies also demonstrated non-significant differences between shaving technique and nerve resection in PTC patients’ RFS and disease specific survival, while shaving technique preserved better RLN function (13,32,46). As for postoperative hypoparathyroidism, non-statistical significances were detected between those with or without RLN invasion, suggested that standardized procedures and abundant experience are the keys to preserve intact parathyroid function when dealing with RLN involvement during the surgery. Management of the compromised RLN is a complex task, requiring synthesis of multiple elements, for instance preoperative RLN examination, intraoperative surgical anatomic information, disease characteristics, and relevant patient factors. We believe tumor shaving for carefully selected cases should be considered to maintain vocal cords mobility with oncological safety; if it is difficult to preserve the nerve during complete removal of the tumor, it is desirable to adopt the reconstruction procedure simultaneously.

There are some inherent limitations in this study. Firstly, potential sample bias may occur which could attribute to the retrospective nature of this single-center research. Secondly, the limited time span of follow-up may not sufficient to observe all disease recurrence events. Improved information on the molecular alterations should be included as they are intrinsic drivers of various aggressive behaviors of PTC. Finally, a prospective study with larger numbers of patients is needed to fully evaluate the oncologic safety of RLN shaving technique.

In conclusion, we confirmed the rarity of RLN invasion in patients with PTC ≤ 1 cm in this study. Preoperative VCP, gross extrathyroidal extension, larger tumor size and tumor on the dorsal side of thyroid are predictive factors of RLN invasion. The tumor shaving technique showed superiority in preserving nerve function without increasing recurrent risk when carefully performed. Special attentions should be paid for disease recurrence when RLN invasion accompanied by upper-aerodigestive tract invasion, lateral neck LNM or BRAF V600E mutation.
